# Effects of STC1 overexpression on tumorigenicity and metabolism of hepatocellular carcinoma

**DOI:** 10.18632/oncotarget.23566

**Published:** 2017-12-21

**Authors:** Cherry CT Leung, Chris KC Wong

**Affiliations:** ^1^ Department of Biology, Hong Kong Baptist University, Hong Kong SAR, China

**Keywords:** xenograft, tumor mass, boyden chamber, rpS6, AMPK

## Abstract

Stanniocalcin-1 (STC1) is a paracrine factor associated with inflammation and carcinogenesis. Using clinicopathological data, we previously reported that a greater expression of STC1 in hepatocellular carcinoma (HCC) was significantly correlated with smaller tumor size. The underlying mechanism on the correlation is not known. In this study, using a metastatic HCC cell-line (MHCC-97L, P) and lentiviral vector mediated-STC1 overexpression, the inoculation of STC1-overexpressing MHCC-97L (S1) cells in a nude mice xenograft model demonstrated reductions in tumor mass and volume. As compared with P cells, S1 cells exhibited epithelial phenotype with significantly lower plating efficiency and reduced migratory and proliferative potential. Using coulter counter for cell-sizing, S1 cells (17.6 μm) were significantly smaller than P cells (19.6 μm). Western blot analysis revealed that S1 cells exhibited reduced expression level of phosphorylated ribosomal protein S6 (p-rpS6). Moreover, an inhibition of the upstream kinase p70^S6K^ was evident with the dephosphorylation of Thr389 in the linker domain of the kinase. The inhibition of p70^S6K^/p-rpS6 pathway was accompanied with reduced cellular ATP level and increase of p-AMPK in S1 cells. Significantly lower rates of glycolysis and extracellular O_2_ consumption in S1 cells exhibited a lower cellular energy status. Since a faster rate of ATP production is essential to support cancer growth and metastasis, the present study identified the effect of STC1-overexpression on reducing energy metabolism, leading to an activation of AMPK pathway but an inhibition of p70^S6K^/p-rpS6 signaling to reduce tumor growth.

## INTRODUCTION

Stanniocalcin-1 (STC1) is a fish hypocalcemic hormone that targets on fish gills to inhibit Ca^2+^-transport. The mammalian homolog of the hormone was cloned in the screening of cancer-related gene [1] and was identified in the transcriptional profiling of serum-starved human fibroblasts [2]. The data implicated that the hormone is likely involved in growth-related processes and metabolism. Interestingly, an early STC1-transgenic mouse model showed dwarf phenotypes [3]. In mammals, the hormone is broadly expressed in various body tissues and is recognized as a paracrine/autocrine factors. Recent works have revealed the association of STC1 expressions with various pathological mechanisms and diseases phenotypes, in particular to inflammation, tumor growth and metastasis [4, 5]. Significant upregulations of STC1 were reported in tumors under hypoxic or oxidative stress [6, 7]. Considerable numbers of studies have also shown differential expressions of STC1 in paired human tumor and normal tissues. Increased STC1 expression was mostly detected in human tumor samples of colorectal cancers and hepatocellular carcinomas (HCC) [8, 9], non-small cell lung cancer [10], ovarian cancer [11], breast carcinoma [12–14] and leukemia [15]. Many laboratory experiments have revealed that STC1 expression was associated with tumorigenesis in renal [16], breast and ovarian cancers [11, 17]. However, the association of greater expressions of STC1 in tumors with either poor or good prognosis is still not conclusive [5].

Current evidence supports the association between STC1 expression and tumorigenesis. The involvement of STC1 in the processes of tumor growth, epithelial-mesenchymal transition (EMT) and apoptosis in various types of cancer were suggested [11, 16, 18–23]. Our previous study using clinicopathological data of HCC showed significantly greater expression of STC1 in tumors versus the paired normal samples [24]. However a negative correlation (*p* = 0.008, 216 patient samples) of STC1 expression with tumor size was observed. Using STC1-overexpressing metastatic HCC cell-line (MHCC-97L) in the study, the negative correlation was confirmed. However, the underlying molecular mechanism on how STC1 reduced tumor masses is not clear. In fact, the dwarf phenotypes in STC1-overexpressing transgenic mice may provide some clues on the role of STC1. Intriguingly, transmission electron microscopy of the transgenic mouse tissues revealed enlargement of mitochondria [25]. The observation suggested that STC1 may target on mitochondria to affect cell metabolism. Latter studies in the characterization of STC1 receptor suggested a functional role of STC1 to uncouple the process of oxidative phosphorylation [26, 27] and to activate mitochondrial antioxidant pathways [28]. With the benefit of hindsight, in this study lentiviral-based overexpression approach was applied to monitor tumor growth in nude mice xenograft and to characterize *in vitro* functional implications of STC1. Using the metastatic HCC cell-line (MHCC-97L), biochemical and molecular pathway analyses were performed to elucidate the effects of STC1-overexpression on the epithelial phenotype, tumorigenicity and metabolism of the cells.

## RESULTS

### Effect of STC1-overexpression on cell phenotype

**Figure 1:**
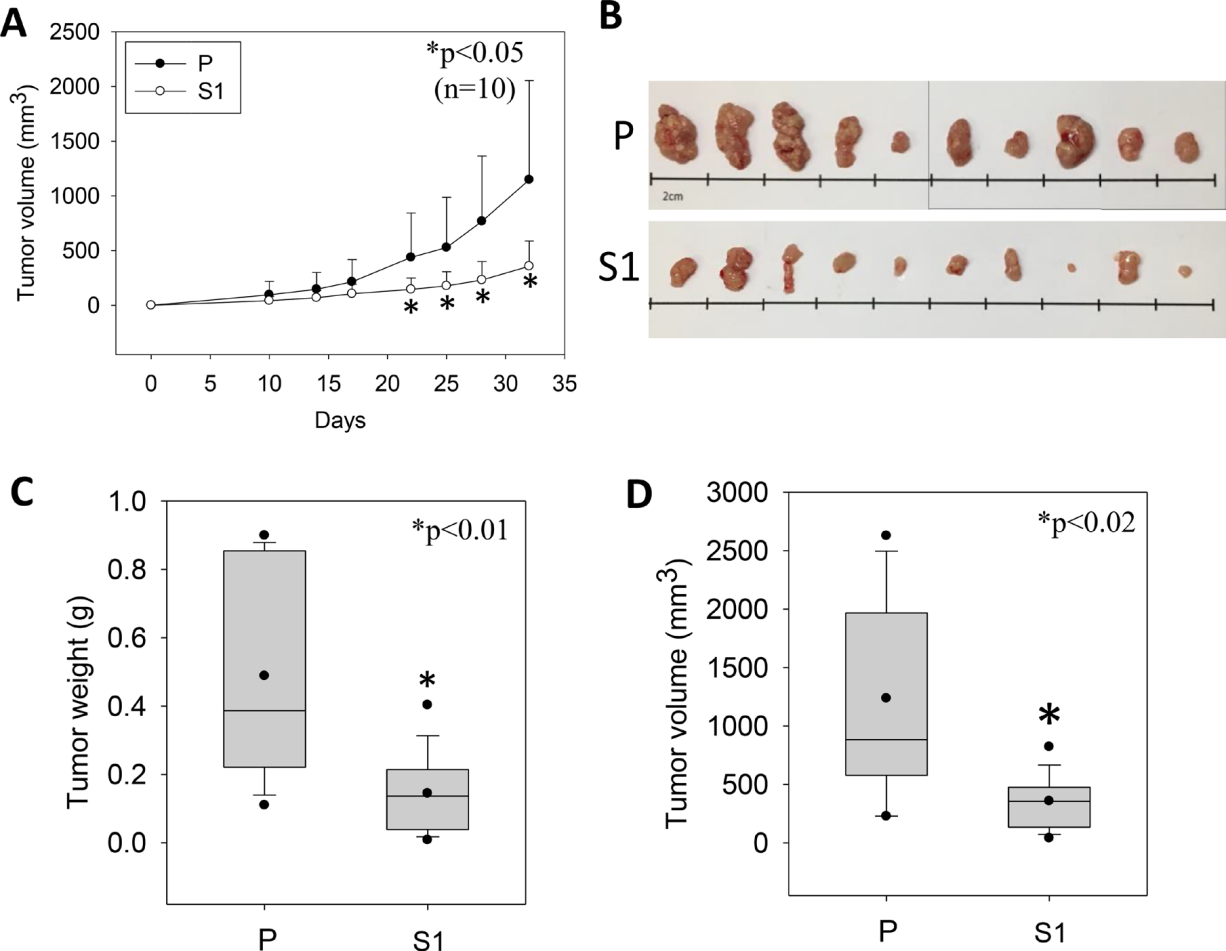
The growth of the human metastatic hepatocellular carcinoma cell-line (MHCC-97L, P) and lentiviral vector mediated-STC1 overexpressing MHCC-97L (S1) in immunodeficient mice (**A**) The measurement of tumor volumes of P- and S1-derived xenografts in mice against the days of inoculation. From day 22 onwards until the end of the experiment (day 33), significant smaller tumor volumes were recorded in S1-derived xenografts (*n* = 10, ^*^p < 0.05). (**B**) The photomicrographs shows the isolated P- and S-derived xenografts. Panels (**C**) and (**D**) show the distributions of tumor weights and volumes, respectively. Significant lesser tumor weights and volumes were measured in S1-derived xenografts.

**Figure 2:**
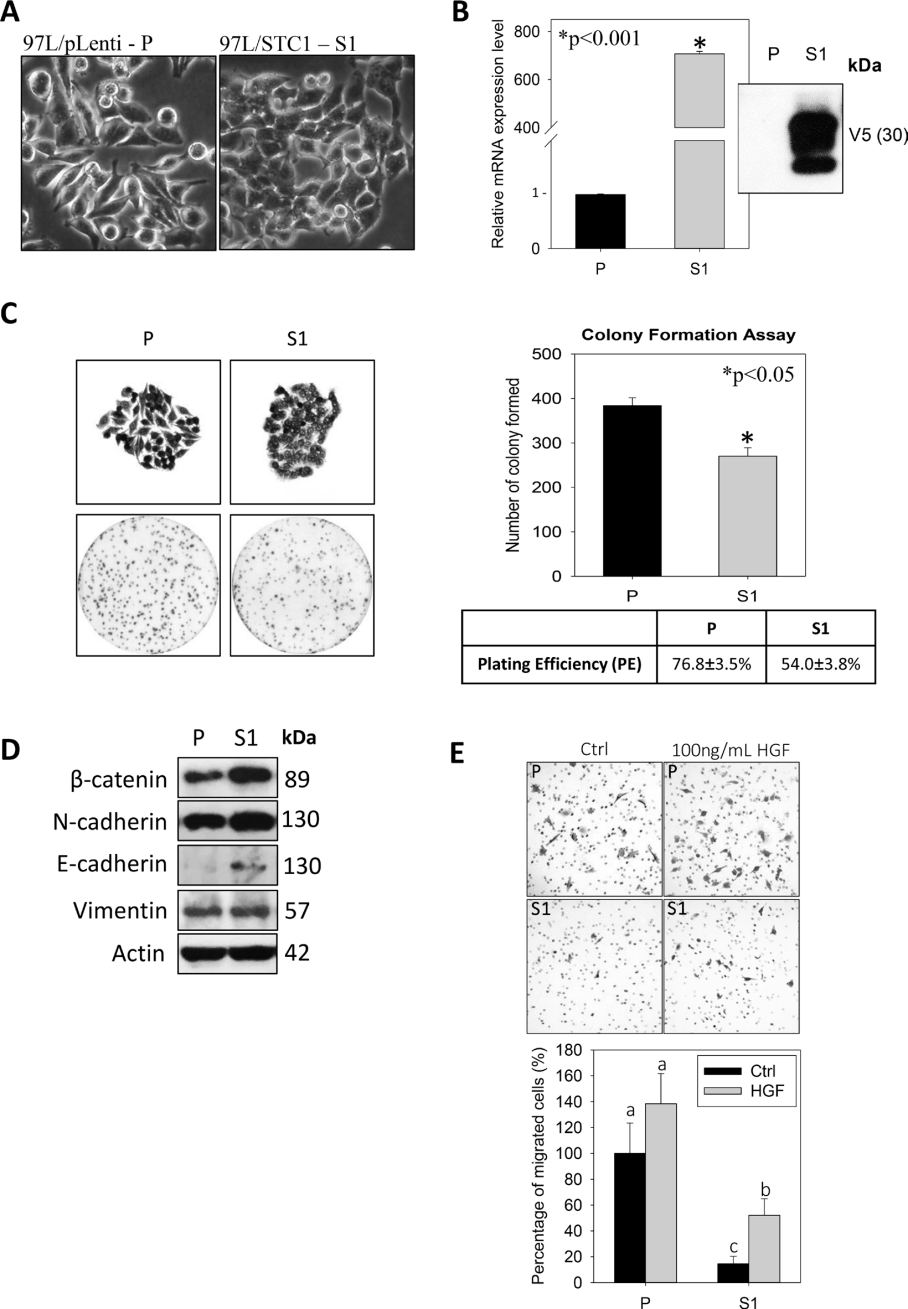
Tumorigenicity-phenotype of the human metastatic hepatocellular carcinoma cell-line (MHCC-97L, P) and lentiviral vector mediated-STC1 overexpressing MHCC-97L (S1) in culture (**A**) Light micrographs show the elongated fibroblast-like and polygonal phenotype of P and S1 cells, respectively. (**B**) The validation of the mRNA and protein expression levels of STC1 in S1 cells using real-time PCR and western blotting. (**C**) Representative images of cell colonies and plate-reads of P and S1 cells (left panel). The statistical analysis of the colony form and plating efficiency (right panel). (**D**) Western blot analysis shows the expression levels of epithelial-mesenchymal transition (EMT) proteins in P and S1 cells. Significant greater expression levels of β-catenin and E-cadherin were observed in S1 cells. (**E**) Boyden chamber assay shows that the migratory responses of S1 was significant lesser than P cells with or without the addition of the hepatocyte growth factor (HGF, 100 ng/ml). Bars with the same letter are not significantly different according to the results of one-way ANOVA followed by Duncan's multiple ranges tests (*p* < 0.05).

STC1 overexpression was established in the metastatic human hepatocellular carcinoma (MHCC-97L) using lentivirus approach. The S1-derived xenografts in immunodeficient mice exhibited significantly lower tumor volumes starting from day 22 to 33 of the post-inoculation (Figure 1A). On day 33, the dissected tumor masses from the mice inoculated with wild-type MHCC-97L (P) or MHCC-97L-STC1 (S1) cells were shown (Figure 1B). *In vitro* measurement of the tumor sizes showed consistent observation as the *in vivo* data in which the tumor weights and volumes were significantly lesser in the S1 xenografts (Figure 1C–1D). When comparing the cell morphology between P and S1 cells, P cells showed elongated fibroblast-like phenotype (Figure 2A). The S1 cells however exhibited the morphology to be more polygonal in shape. Quantitative real-time PCR and western blot analysis showed a remarkable increase in the levels of STC1 transcript and protein in S1 cells (Figure 2B), illustrating the high efficacy of the viral infection. Colony formation assay showed significantly lower plating efficiency of S1 cells (Figure 2C). Microscopic examination of the cell colonies also illustrated that S1 cells were more tightly packed as compared with the P cells. Western blot analysis showed that the S1 cells had greater expression levels of β-catenin, N-cadherin and E-cadherin (Figure 2D). Boyden chamber assay revealed that the migratory responses of S1 was significantly lesser than P cells, with or without the addition of the hepatocyte growth factor (HGF) (Figure 2E).

**Figure 3:**
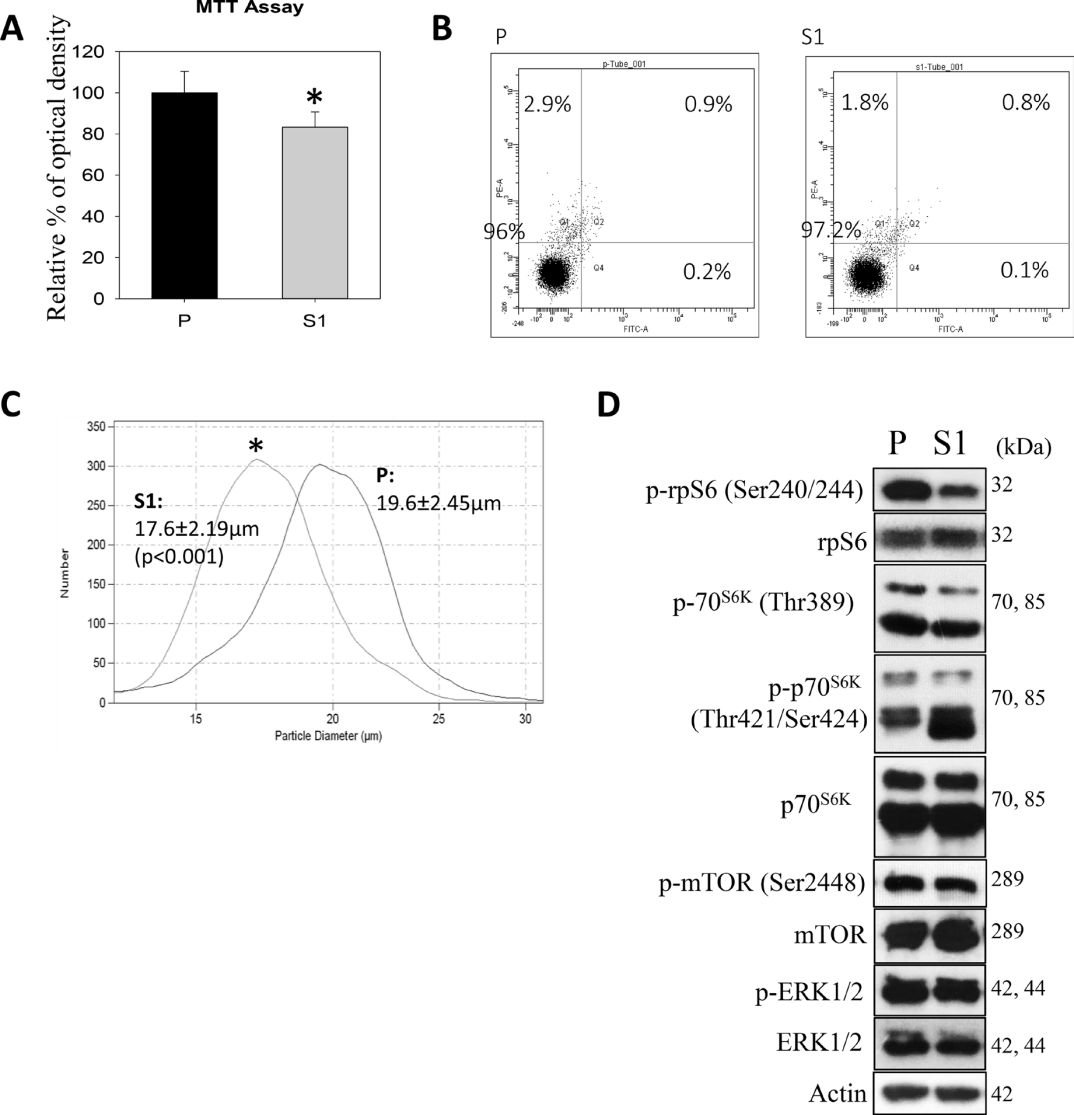
Cytological and molecular characterization of the human metastatic hepatocellular carcinoma cell-line (MHCC-97L, P) and lentiviral vector mediated-STC1 overexpressing MHCC-97L (S1) in culture (**A**) MTT assay shows significant lower proliferative rate of S1 cells. (**B**) Flow cytometry (annexin V-PI) shows no significant differences in the percentages of apoptotic or PI^+^ populations of P and S1 cells. (**C**) Coulter counter for cell-sizing shows S1 exhibited significant smaller cell size as compared with P cell population. (**D**) Western blot analysis show the expression levels of phosphorylated protein kinases in P and S1 cells. Significant lesser levels of p-70^S6K^(Thr 389)/p-rpS6 were observed in S1 cells.

**Figure 4:**
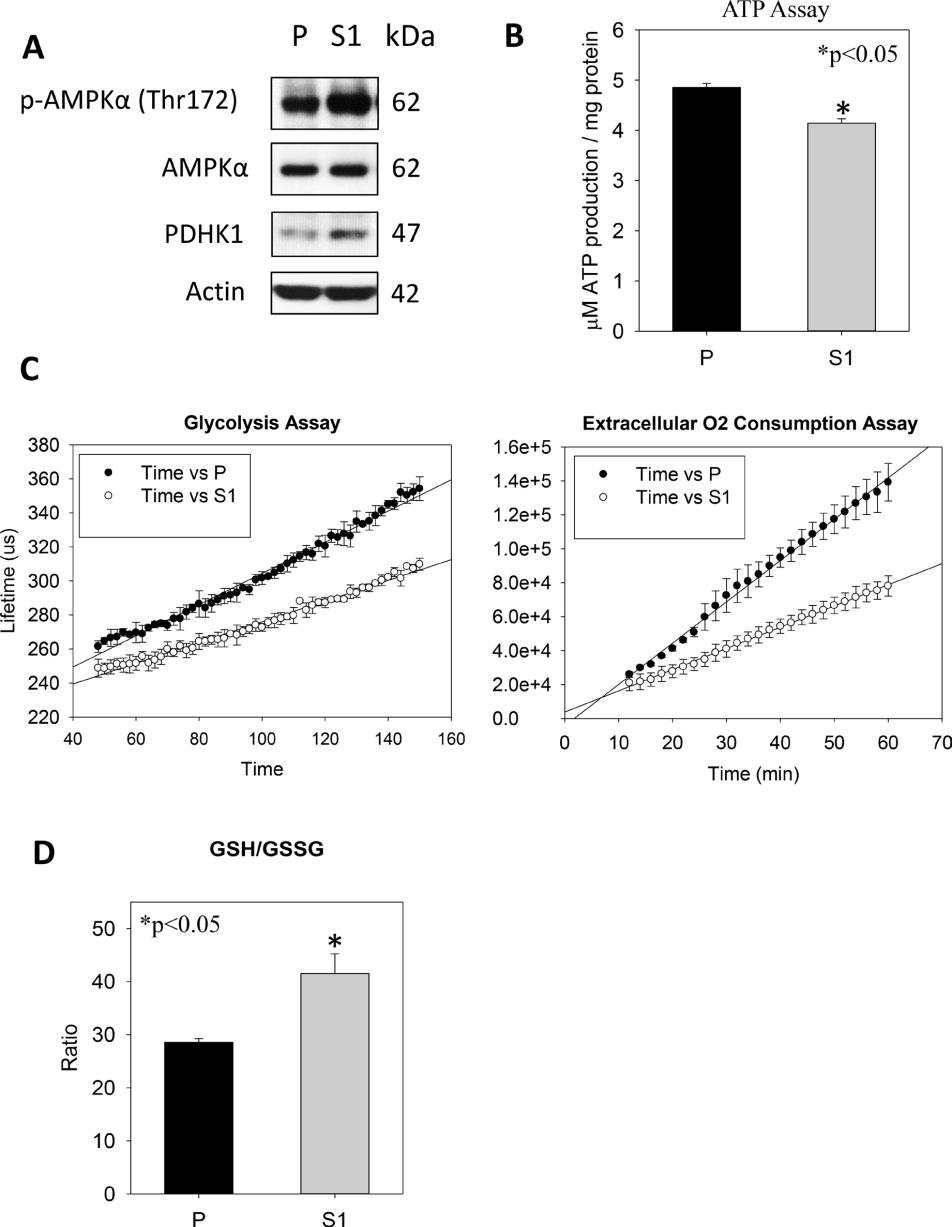
Metabolism effects of STC1 overexpression on MHCC-97L (S1) in culture (**A**) Western blot analysis shows significant greater levels of p-AMPK and PDHK1 in S1 cells. (**B**) ATP determination shows significant lower cellular ATP levels in S1 cells. (**C**) Glycolysis (left panel) and extracellular O_2_ consumption (right panel) assays show significant lower rates of metabolic processes in S1 cells. (**D**) A greater GSH/GSSG ratio was measured in S1 cells.

In cytological and molecular characterization, MTT data indicated significantly lower proliferation of S1 cells (Figure 3A). Annexin V-PI flow cytometry revealed no significant differences in the percentages of apoptotic or PI^+^ cell populations (Figure 3B). Interestingly, the use of a Coulter counter for cell-sizing showed significantly smaller cell size of S1 as compared with P cell population (Figure 3C). S1 cells had the average cell diameter of 17.6 ± 2.19μm, which were significantly smaller (*p* < 0.001) than P cells (19.6 ± 2.45μm). Since the phosphorylation of ribosomal protein (rp) S6 is an important determinant of cell size, we measured the cellular levels of the phosphorylated (Ser240/244) and total rpS6 using western blotting. Figure 3D showed that S1 cells had significantly lower phosphorylation level of rpS6 (p-rpS6) as compared with P cells. Moreover a significant reduction in the phosphorylation of the upstream regulatory kinase p70^S6K^ (Thr 389) in S1 cells was observed. On the other hand, an increase in the phosphorylation of p70^S6K^ (Thr421/Ser424) at the C-terminal auto-inhibitory pseudosubstrate domain was evident. Although the phosphorylation at the pseudo-region was thought to activate p70^S6K^, Le and co-workers demonstrated that the phosphorylation is not necessarily concordant with the activation [29]. Instead, paclitaxel-induced phosphorylation at Thr421/Ser424 was found to reduce the activity of p70S6K. Therefore, the changes in the phosphorylation patterns at Thr389 at the linker domain of p70^S6K^ in S1 cells supported the notion that the smaller cell size phenotype was linked to the inactivation of the p70^S6K^-p-rpS6 pathway. Nonetheless, there was no noticeable change in the phosphorylation of mTOR (Ser2448), an upstream kinase of p70^S6K^. This observation prompted us to investigate another functionally-related kinase, AMP-activated protein kinase (AMPK), which negatively regulates mTOR/p70^S6K^ pathway [30]. Western blot analysis showed a significantly greater level of pAMPKα (Thr172) in S1 cells (Figure 4A). Cellular ATP level (Figure 4B), glycolytic and extracellular O_2_ consumption rates (Figure 4C) were significantly lower in S1 cells (*p* < 0.05, fit-regression analysis, Minitab). In addition, a significantly greater expression level of pyruvate dehydrogenase kinase (PDHK1) (Figure 4A), which negatively regulates pyruvate dehydrogenase (PDH) flux to reduce mitochondria-derived ATP [31] was noted in S1 cells. The decreased cellular metabolism in mitochondrial respiratory pathway was accompanied with a significantly greater GSH/GSSG ratio (Figure 4D).

## DISCUSSION

Our previous study showed that STC1 was upregulated in the tumor tissues from the analysis of clinical data of 216 HCC patients [24]. The observation implied the greater expression of STC1 being associated with poor prognostic outcome. However the same set of date showed tumors with greater expression level of STC1 (tumor/normal ≥ 2) being significantly smaller (*p* = 0.008) than the samples with lower STC1 (tumor/normal < 2). Using HCC cell-line analysis, we showed the inhibitory actions of STC1 on the pro-migratory effects of IL-6/IL-8, the growth of tumor spheroids in culture and the development of tumor mass in nude mice model [24]. The underlying mechanisms of a greater STC1 expression in the smaller tumor mass of HCC is not clear.

In the present study, using the metastatic hepatocellular carcinoma (MHCC-97L, P) and STC1-lentivirus overexpressed cells (MHCC-97L-STC1, S1), we identified that STC1-overexpression transformed P cells from fibroblast to epithelial phenotype (S1) in culture. Greater expression levels of E-cadherin and β-catenin were evident in S1 cells. Although β-catenin is able to form nuclear complexes with high mobility group transcription factors (the lymphoid enhancer binding factor (LEF)-1/T cell factor (TCF) family) to induce epithelial-mesenchymal transition, the upregulation of E-cadherin is known to inhibit β-catenin transcriptional activity [32, 33]. In addition β-catenin is part of a protein complex that forms adherens junctions for E-cadherin mediated cell adhesion for the maintenance of epithelial cell layers [34]. Thus, the greater expression level of E-cadherin in S1 cells might lead to the epithelial phenotype. In fact, the supporting role of STC1 in the process of re-epithelization in human keratinocytes was demonstrated [35]. Moreover, in the present study the epithelial phenotype of S1 cells was observed in the colony formation assay, with significantly lower plating efficiency.

Using Coulter counter analysis, S1 cells showed significantly smaller cell size. The smaller cell size was associated with lower level of ribosomal protein S6 phosphorylation (p-rpS6), reduced cellular ATP contents and greater level of phosphorylated AMPK. The phosphorylation of rpS6 is known to be a positive regulator of cell size [36] and is regulated by the upstream kinase p70^S6K^ [37] and mitogen activated protein kinase [38]. Our data showed the lower phosphorylation of p70^S6K^ in S1 cells while no significant changes in the levels of phosphorylated ERK1/2. Indeed, p70^S6K-/−^ mice exhibited smaller cell and animal sizes [39]. Likewise, the overexpression of human STC1 in mice showed a growth retardation phenotype [3, 40] in which a possible role of STC1 in mitochondrial function and cell metabolism was suggested [25]. To address the role of STC1 in cell metabolism, the experiments for the determination of cellular ATP level, glycolytic flux and extracellular O_2_ consumption were conducted. S1 cells showed significantly lower cellular ATP levels, which was associated with lower rates of glycolysis and extracellular O_2_ consumption. The reduced mitochondrial respiration in S1 cells might result in a lower cellular level of reactive oxygen species which was revealed by a greater ratio of GSH/GSSG in the cells [41]. Our observation also aligned with the finding of other studies. The function of STC1 was found to uncouple the process of oxidative phosphorylation via an increased expression of mitochondrial UCP2 [42] to reduce ATP synthesis [26, 27]. Moreover, the effect of STC1 on the stimulation of gluconeogenesis in rat and fish kidneys was suggested [43], which is the endergonic process to consume cellular ATP. The reduced cellular ATP levels could be perceived by the key regulator in the cellular energy metabolism, AMPK a sensor of ADP/AMP/ATP ratio [30]. Presumably, AMPK was activated under the low cellular ATP content in S1 cells, to block the phosphorylation of p70^S6K^ [44]. Therefore, the greater level of phosphorylated AMPK (p-AMPK, Thr172) in S1 cells may explain the lower level of p70^S6K^ (Thr 389) in S1 cells, even though the levels of p-mTOR were comparable between P and S1 cells. Intriguingly, Pan and coworkers reported that the high STC1 expression in a transgenic mice model was associated with a greater AMPK activity as compared with the corresponding wild-type and STC1 knockout animals [45]. The data supported the functional association between STC1 and AMPK activity. Taken together, the literatures and our data support the observations of the smaller tumor mass of S1 cells in nude mice and the clinicopathological analysis in our previous study [24]. S1 cells exhibited lower cellular ATP levels and reduced p70^S6K^/p-rpS6 signals, which were the possible causes of smaller tumor mass in the nude mice.

In summary, the present study showed the smaller tumor xenografts in nude mice and cell size derived from STC1-overexpressed metastatic human HCC-97L cells. Follow-up experimental analysis underlined that S1 cells exhibited epithelial phenotype, low plating efficiency and reduced migratory potential. Western blotting analysis illustrated the downregulation of p70^S6K^/p-rpS6 pathway but an upregulation of AMPK pathway, underpinning the molecular pathways associated with the smaller cell size phenotype. The significantly lower glycolysis, O_2_ consumption and ATP production in the STC1-overexpressed cells attributed to the reduction of cellular energy metabolism.

## MATERIALS AND METHODS

### Cell culture

Metastatic hepatocellular carcinoma (MHCC-97L) and human embryonic kidney cell line HEK293FT were cultured in a high-glucose Dulbecco's modified Eagle's medium (DMEM) (Gibco; Life Technologies) supplemented with 10 % heat-inactivated fetal bovine serum (Gibco; Life Technologies) and antibiotics (25 U/mL penicillin and 25 μg/mL streptomycin) (Life Technologies).

### Lentiviral overexpression of STC1 in MHCC-97L cells

Human STC1 cDNA that encodes the wild-type full-length protein was cloned into pENTR/SD/D-TOPO (Life Technologies) according to the manufacturer's instructions. STC1-insert was then sub-cloned into the expression vector pLenti6.3/TO/V5-DEST (Life Technologies) using Gateway LR Clonase II Plus Enzyme Mix (Life Technologies). HEK293FT cells were seeded into 100 mm dishes overnight, followed by co-transfection with the ViralPower Packaging Mix and pLenti6.3/TO/V5-DEST-STC1 or pLenti6.3/TO/V5-DEST using Lipofectamine 2000 (Invitrogen). Supernatants containing virus were collected after 48 hr and stored at −80°C. MHCC-97L (P) were seeded into 6-well plate overnight, then transduced with lentiviral particles and 6 μg/mL polybrene (Sigma-Aldrich) for 24 hr. The stably infected cells (S1) were selected using 4 μg/mL blasticidin (Life Technologies) for over 2 weeks. STC1 overexpression were verified by qPCR and western blotting.

### Xenograft animal model

Two hundred microliters of P or S1 cells (2 × 10^6^ cells) were subcutaneously inoculated in the right frank of 6-week-old male BALB-c nude mice (*n* = 10) using 29-gauge needles. Tumor volumes were measured twice per week and calculated by the equation [(width^2^ × length)/2]. When the average volume of inoculated tumor in the control group was about 700 mm^3^, all the mice were sacrificed. In this experiment on day 32, the tumor samples were collected and weighed.

### Colony formation assay

P and S1 cells were seeded in 6-well plates (500 cells/well, *n* = 6, in a complete DMEM high glucose medium). After 10-day of the incubation, cells were fixed with ice-cold methanol for 10 min at −20°C, and stained with 0.5 % crystal violet (Farco Chemical Supplies) in 20 % methanol at room temperature for 10 min. Colony images were captured using light microscopy and counted by ImageJ.

### Boyden chamber-based cell migration assay

Migration assay was performed in 24-well cell culture inserts (Falcon) with 8 μm pore size membrane. P and S1 cells were trypsinized from 100 mm plates, then washed with a serum-free DMEM twice. The cells were seeded into the inserts in the serum-free medium with or without 100 ng/mL hepatocyte growth factor (HGF, Gibco), whereas the lower chambers of the wells were filled by the medium containing 10 % FBS. After 24 hr incubation at 37°C, the cells on the top side of the insert membranes were removed by cotton swabs. The cells migrated to the bottom side of the insert membrane were fixed with ice-cold methanol at −20°C for 10 min, then stained with 0.5% crystal violet (Farco Chemical Supplies) in 20% methanol at room temperature for 10 min. Migrated cells were counted and captured using light microscopy.

### Western blot analysis

Cells were lysed in cold radioimmunoprecipitation assay (RIPA) buffer (50 mM Tris-HCL, pH7.4, 50 mM NaCl, 2 mM EDTA, 1 % nonidet P-40, and 0.1 % SDS) and sonicated by Bioruptor Plus (Diagenode), followed by centrifugation at 12,000 g for 10 min at 4°C. Supernatant was collected, and protein concentration was measured using DC Protein Assay Kit II (Bio-Rad). Absorbance at 750 nm was determined using a microplate reader (Bio Tek, ELX800). Protein lysates were resolved in SDS-PAGE and transferred onto PVDF membranes (Bio-Rad). The membrane was blocked in 5% non-fat milk in PBST for 1 hr and probed with primary antibody (Supplementary Table 1) followed by HRP-conjugated secondary antibody (Bio-Rad Pacific Ltd). Specific bands were visualized by WESTSAVE Up (AbFrontier).

### MTT assay

Cells were seeded in quadruplicate in 96-well plates. After an overnight incubation, the medium was removed and was replaced by MTT solution (250 μg/mL in PBS, Molecular Probes). The cells were incubated in a 37°C incubator for 4 hr. Insoluble formazan salts were then dissolved in DMSO (Sigma). Absorbances at 540 nm and 690 nm were detected by the microplate reader.

### Flow cytometry annexin/PI

Cell suspension was washed with cold PBS twice according to the manufacturer's instruction (BD Pharmingen), then resuspended in 1× binding buffer (1 × 10^6^ cells/mL). One hundred microliters of cells were then transferred to a 5 mL flow tube, followed by the addition of Annexin V-FITC and PI, and incubated for 15 min in dark at room temperature. Additional 400 μl 1× binding buffer was added before flow cytometry analysis.

### Coulter counter cell-sizing

Cell suspension was washed and resuspended in 10 mL PBS. Cell size was determined by Coulter Counter (Beckman) with an aperture diameter of 100 μm. Five hundred microliter of cell suspension were analyzed. Ten thousand cells were counted for each measurement.

### Total RNA extraction and real-time PCR

Total RNA was extracted by TRIZOL Reagent (Gibco/BRL) according to the manufacturer's instructions. The A _260_/A_280_ ratio of total cellular RNA was > 1.8 and was used for cDNA synthesis using the High Capacity RNA-to-cDNA Master Mix (Applied Biosystems, Foster City, CA, USA). Real-time PCR was conducted using the StepOne real-time PCR detection system and Fast SYBR Green Master Mix (Applied Biosystems). The primer sequences of human STC1 (5′- TGAGGCGGAGCAGAATGACT −3′, 5′-CAGGTGGAGTTTTCCAGGCAT-3′) and actin (5′-GACTACCTCATGAAGATCCT CACC-3′, 5′-TCTCC TTAATGTCACGCACGATT-3′) were used. Gene expression levels were calculated by the ‘^ΔΔ^Ct method’ and were normalized using actin transcript levels.

### ATP determination

Cells were seeded in quadruplicate in 24-well plates. After an overnight incubation, cells were lysed and centrifuged at 10,000 g for 2 min at 4°C. Supernatant was mixed with an ATP standard reaction solution (Molecular Probes) according to the manufacturer's instructions and luminescence was measured (VICTOR X4, Perkin Elmer). Cellular ATP levels were normalized by the protein concentrations.

### Glycolysis assay

Cells were seeded in triplicate in 96-well plates. After an overnight incubation, the culture medium was removed and the cells were washed with a respiration buffer according to the manufacturer's instruction (Abcam). A glycolysis reagent was added to each sample-well. The fluorescent signal was measured by dual-read time-resolved fluorescence at Ex_340nm_ and Em_615nm_ (VICTOR X4, Perkin Elmer) every 2 min for 2 hr at 37°C.

### Extracellular oxygen consumption assay

Cells were seeded in triplicate in 96-well plates. After an overnight incubation, medium was replaced by a fresh culture medium, an extracellular O_2_ consumption reagent was added to each sample-well according to the manufacturer's instruction (Abcam). The wells were promptly sealed with mineral oil. Fluorescent signals were measured by dual-read time-resolved fluorescence at Ex_340nm_ and Em_642nm_ (VICTOR X4, Perkin Elmer) every 2 min for 2 hr at 37°C.

### GSH/GSSG-Glo assay

Cells were seeded in quadruplicate in 96-well plates. After an overnight incubation, cells were lysed with the total glutathione lysis or oxidized glutathione lysis reagents, followed by an addition of a luciferin detection reagent according to the manufacturer's instructions (Promega). Luminescence was measured (VICTOR X4, Perkin Elmer), and the ratio of total glutathione to oxidized glutathione was calculated.

### Statistical analysis

Statistical analysis was conducted using SigmaStat version 3.5. Data were evaluated by the Student's *t*-test or one-way analysis of variance (ANOVA) followed by Duncan's multiple range test. All data are presented as statistical mean ± SD. A *p* value < 0.05 was used as the cutoff for statistical significance.

## SUPPLEMENTARY MATERIALS TABLE



## References

[1] Chang AC, Janosi J, Hulsbeek M, de Jong D, Jeffrey KJ, Noble JR, Reddel RR (1995). A novel human cDNA highly homologous to the fish hormone stanniocalcin. Mol Cell Endocrinol.

[2] Iyer VR, Eisen MB, Ross DT, Schuler G, Moore T, Lee JC, Trent JM, Staudt LM, Hudson J, Boguski MS, Lashkari D, Shalon D, Botstein D (1999). The transcriptional program in the response of human fibroblasts to serum. Science.

[3] Varghese R, Gagliardi AD, Bialek PE, Yee SP, Wagner GF, DiMattia GE (2002). Overexpression of human stanniocalcin affects growth and reproduction in transgenic mice. Endocrinology.

[4] Chang AC, Jellinek DA, Reddel RR (2003). Mammalian stanniocalcins and cancer. Endocr Relat Cancer.

[5] Yeung BH, Law AY, Wong CK (2012). Evolution and roles of stanniocalcin. Mol Cell Endocrinol.

[6] Nguyen A, Chang AC, Reddel RR (2009). Stanniocalcin-1 acts in a negative feedback loop in the prosurvival ERK1/2 signaling pathway during oxidative stress. Oncogene.

[7] Yeung HY, Lai KP, Chan HY, Mak NK, Wagner GF, Wong CK (2005). Hypoxia-inducible factor-1-mediated activation of stanniocalcin-1 in human cancer cells. Endocrinology.

[8] Fujiwara Y, Sugita Y, Nakamori S, Miyamoto A, Shiozaki K, Nagano H, Sakon M, Monden M (2000). Assessment of Stanniocalcin-1 mRNA as a molecular marker for micrometastases of various human cancers. Int J Oncol.

[9] Tamura S, Oshima T, Yoshihara K, Kanazawa A, Yamada T, Inagaki D, Sato T, Yamamoto N, Shiozawa M, Morinaga S, Akaike M, Kunisaki C, Tanaka K (2011). Clinical significance of STC1 gene expression in patients with colorectal cancer. Anticancer Res.

[10] Du YZ, Gu XH, Li L, Gao F (2011). The diagnostic value of circulating stanniocalcin-1 mRNA in non-small cell lung cancer. J Surg Oncol.

[11] Liu G, Yang G, Chang B, Mercado-Uribe I, Huang M, Zheng J, Bast RC, Lin SH, Liu J (2010). Stanniocalcin 1 and ovarian tumorigenesis. J Natl Cancer Inst.

[12] Joensuu K, Heikkila P, Andersson LC (2008). Tumor dormancy: elevated expression of stanniocalcins in late relapsing breast cancer. Cancer Lett.

[13] McCudden CR, Majewski A, Chakrabarti S, Wagner GF (2004). Co-localization of stanniocalcin-1 ligand and receptor in human breast carcinomas. Mol Cell Endocrinol.

[14] Wascher RA, Huynh KT, Giuliano AE, Hansen NM, Singer FR, Elashoff D, Hoon DS (2003). Stanniocalcin-1: a novel molecular blood and bone marrow marker for human breast cancer. Clin Cancer Res.

[15] Tohmiya Y, Koide Y, Fujimaki S, Harigae H, Funato T, Kaku M, Ishii T, Munakata Y, Kameoka J, Sasaki T (2004). Stanniocalcin-1 as a novel marker to detect minimal residual disease of human leukemia. Tohoku J Exp Med.

[16] Ma X, Gu L, Li H, Gao Y, Li X, Shen D, Gong H, Li S, Niu S, Zhang Y, Fan Y, Huang Q, Lyu X (2015). Hypoxia-induced overexpression of stanniocalcin-1 is associated with the metastasis of early stage clear cell renal cell carcinoma. J Transl Med.

[17] Welcsh PL, Lee MK, Gonzalez-Hernandez RM, Black DJ, Mahadevappa M, Swisher EM, Warrington JA, King MC (2002). BRCA1 transcriptionally regulates genes involved in breast tumorigenesis. Proc Natl Acad Sci U S A.

[18] Chang AC, Doherty J, Huschtscha LI, Redvers R, Restall C, Reddel RR, Anderson RL (2015). STC1 expression is associated with tumor growth and metastasis in breast cancer. Clin Exp Metastasis.

[19] Ching LY, Yeung BH, Wong CK (2012). Synergistic effect of p53 on TSA-induced stanniocalcin 1 expression in human nasopharyngeal carcinoma cells, CNE2. J Mol Endocrinol.

[20] Lai KP, Law AY, Yeung HY, Lee LS, Wagner GF, Wong CK (2007). Induction of stanniocalcin-1 expression in apoptotic human nasopharyngeal cancer cells by p53. Biochem Biophys Res Commun.

[21] Law AY, Lai KP, Lui WC, Wan HT, Wong CK (2008). Histone deacetylase inhibitor-induced cellular apoptosis involves stanniocalcin-1 activation. Exp Cell Res.

[22] Ohkouchi S, Block GJ, Katsha AM, Kanehira M, Ebina M, Kikuchi T, Saijo Y, Nukiwa T, Prockop DJ (2012). Mesenchymal stromal cells protect cancer cells from ROS-induced apoptosis and enhance the Warburg effect by secreting STC1. Mol Ther.

[23] Pena C, Cespedes MV, Lindh MB, Kiflemariam S, Mezheyeuski A, Edqvist PH, Hagglof C, Birgisson H, Bojmar L, Jirstrom K, Sandstrom P, Olsson E, Veerla S (2013). STC1 expression by cancer-associated fibroblasts drives metastasis of colorectal cancer. Cancer Res.

[24] Yeung BH, Shek FH, Lee NP, Wong CK (2015). Stanniocalcin-1 Reduces Tumor Size in Human Hepatocellular Carcinoma. PLoS One.

[25] Filvaroff EH, Guillet S, Zlot C, Bao M, Ingle G, Steinmetz H, Hoeffel J, Bunting S, Ross J, Carano RA, Powell-Braxton L, Wagner GF, Eckert R (2002). Stanniocalcin 1 alters muscle and bone structure and function in transgenic mice. Endocrinology.

[26] Ellard JP, McCudden CR, Tanega C, James KA, Ratkovic S, Staples JF, Wagner GF (2007). The respiratory effects of stanniocalcin-1 (STC-1) on intact mitochondria and cells: STC-1 uncouples oxidative phosphorylation and its actions are modulated by nucleotide triphosphates. Mol Cell Endocrinol.

[27] McCudden CR, James KA, Hasilo C, Wagner GF (2002). Characterization of mammalian stanniocalcin receptors. Mitochondrial targeting of ligand and receptor for regulation of cellular metabolism. J Biol Chem.

[28] Sheikh-Hamad D (2010). Mammalian stanniocalcin-1 activates mitochondrial antioxidant pathways: new paradigms for regulation of macrophages and endothelium. Am J Physiol Renal Physiol.

[29] Le XF Hittelman WN, Liu J, McWatters A, Li C, Mills GB, Bast RC (2003). Paclitaxel induces inactivation of p70 S6 kinase and phosphorylation of Thr421 and Ser424 via multiple signaling pathways in mitosis. Oncogene.

[30] Hardie DG, Ross FA, Hawley SA (2012). AMPK: a nutrient and energy sensor that maintains energy homeostasis. Nat Rev Mol Cell Biol.

[31] Zhang S, Hulver MW, McMillan RP, Cline MA, Gilbert ER (2014). The pivotal role of pyruvate dehydrogenase kinases in metabolic flexibility. Nutr Metab (Lond).

[32] Stockinger A, Eger A, Wolf J, Beug H, Foisner R (2001). E-cadherin regulates cell growth by modulating proliferation-dependent beta-catenin transcriptional activity. J Cell Biol.

[33] Behrens J, von Kries JP, Kuhl M, Bruhn L, Wedlich D, Grosschedl R, Birchmeier W (1996). Functional interaction of beta-catenin with the transcription factor LEF-1. Nature.

[34] Hartsock A, Nelson WJ (2008). Adherens and tight junctions: structure, function and connections to the actin cytoskeleton. Biochim Biophys Acta.

[35] Yeung BH, Wong CK (2011). Stanniocalcin-1 regulates re-epithelialization in human keratinocytes. PLoS One.

[36] Ruvinsky I, Sharon N, Lerer T, Cohen H, Stolovich-Rain M, Nir T, Dor Y, Zisman P, Meyuhas O (2005). Ribosomal protein S6 phosphorylation is a determinant of cell size and glucose homeostasis. Genes Dev.

[37] Blenis J, Chung J, Erikson E, Alcorta DA, Erikson RL (1991). Distinct mechanisms for the activation of the RSK kinases/MAP2 kinase/pp90rsk and pp70-S6 kinase signaling systems are indicated by inhibition of protein synthesis. Cell Growth Differ.

[38] Pende M, Um SH, Mieulet V, Sticker M, Goss VL, Mestan J, Mueller M, Fumagalli S, Kozma SC, Thomas G (2004). S6K1(−/−)/S6K2(−/−) mice exhibit perinatal lethality and rapamycin-sensitive 5’-terminal oligopyrimidine mRNA translation and reveal a mitogen-activated protein kinase-dependent S6 kinase pathway. Mol Cell Biol.

[39] Shima H, Pende M, Chen Y, Fumagalli S, Thomas G, Kozma SC (1998). Disruption of the p70(s6k)/p85(s6k) gene reveals a small mouse phenotype and a new functional S6 kinase. EMBO J.

[40] Johnston J, Ramos-Valdes Y, Stanton LA, Ladhani S, Beier F, DiMattia GE (2010). Human stanniocalcin-1 or -2 expressed in mice reduces bone size and severely inhibits cranial intramembranous bone growth. Transgenic Res.

[41] Ribas V, Garcia-Ruiz C, Fernandez-Checa JC (2014). Glutathione and mitochondria. Front Pharmacol.

[42] Wang Y, Huang L, Abdelrahim M, Cai Q, Truong A, Bick R, Poindexter B, Sheikh-Hamad D (2009). Stanniocalcin-1 suppresses superoxide generation in macrophages through induction of mitochondrial UCP2. J Leukoc Biol.

[43] Schein V, Kucharski LC, Guerreiro PM, Martins TL, Morgado I, Power DM, Canario AV, da Silva RS (2015). Stanniocalcin 1 effects on the renal gluconeogenesis pathway in rat and fish. Mol Cell Endocrinol.

[44] Krause U, Bertrand L, Hue L (2002). Control of p70 ribosomal protein S6 kinase and acetyl-CoA carboxylase by AMP-activated protein kinase and protein phosphatases in isolated hepatocytes. Eur J Biochem.

[45] Pan JS, Huang L, Belousova T, Lu L, Yang Y, Reddel R, Chang A, Ju H, Dimattia G, Tong Q, Sheikh-Hamad D (2015). Stanniocalcin-1 inhibits renal ischemia/reperfusion injury via an AMP-activated protein kinase-dependent pathway. J Am Soc Nephrol.

