# Supplementary material for: Effects of STC1 overexpression on tumorigenicity and metabolism of hepatocellular carcinoma
Source: Oncotarget. 2017 Dec 21;9(6):6852–61. doi: 10.18632/oncotarget.23566 (PMC5805520; doi:10.18632/oncotarget.23566)
Supplement: Supplementary file 1 [file oncotarget-09-6852-s001.pdf]

Effects of STC1 overexpression on tumorigenicity and metabolism of hepatocellular carcinoma

SUPPLEMENTARY MATERIALS

Supplementary Table 1 : A list of Primary Antibodies

Primary antibody	Company	Cat. No.	Host
β-actin	Sigma-Aldrich	A2228	Mouse
β-catenin	Santa Cruz	sc7963	Mouse
E-cadherin	Santa Cruz	sc-8426	Mouse
Vimentin	Santa Cruz	sc-7558	Goat
N-cadherin	Life Technologies	33-3900	Rabbit
mTOR	Cell Signaling Technology	2983	Rabbit
p-mTOR (Ser2448)	Cell Signaling Technology	2971	Rabbit
p-AMPKα1 (Thr172)	Cell Signaling Technology	2535	Rabbit
AMPKα1	Cell Signaling Technology	5832	Rabbit
70S6K	Cell Signaling Technology	9202	Rabbit
p-70S6K (Thr389)	Cell Signaling Technology	9205	Rabbit
p-70S6K (Thr421/Ser424)	Cell Signaling Technology	9204	Rabbit
rpS6	Cell Signaling Technology	2317	Mouse
p-rpS6 (Ser240, 244)	Cell Signaling Technology	2215	Rabbit
PDHK1	Cell Signaling Technology	3820	Rabbit
V5	Invitrogen	46-0705	Mouse
**Secondary Antibody**			
Anti-Rabbit HRP	Bio-rad	170-6515	Goat
Anti-Mouse HRP	Bio-rad	170-6516	Goat
Anti-Goat HRP	Santa Cruz	sc-2020	Donkey
